# Epiplakin expression in non-melanoma skin cancer: associations with epithelial-mesenchymal transition markers and tumor invasion^؆^

**DOI:** 10.1016/j.abd.2026.501350

**Published:** 2026-04-22

**Authors:** Damla Gül Fındık, Özlem Türelik

**Affiliations:** a Department of Histology and Embryology, Faculty of Medicine, Balıkesir University, Balıkesir, Turkey; b Department of Pathology, Faculty of Medicine, Bilecik Şeyh Edebali University, Bilecik, Turkey

**Keywords:** Basal cell carcinoma, Epithelial-mesenchymal transition, Plakins, Skin neoplasms, Squamous cell carcinoma

## Abstract

**Background/Objectives:**

Epiplakin is a member of the plakin family of proteins involved in cytoskeletal organization, yet its role in skin cancers remains poorly understood. This study aimed to evaluate epiplakin expression in cutaneous skin lesions and to investigate its association with epithelial-mesenchymal transition markers and tumor progression.

**Methods:**

The authors retrospectively analyzed skin specimens from squamous cell carcinomas, basal cell carcinomas, and benign intradermal nevi collected between 2021 and 2025. Histopathological features were assessed, and immunohistochemical analysis of Epiplakin, E-cadherin, and N-cadherin was performed. Epiplakin expression was quantified and correlated with cadherin levels and Breslow thickness. Plakin family protein-protein interaction networks were analyzed using KEGG pathway and GO functional enrichment.

**Results:**

Protein-protein interaction network analysis demonstrated that plakin family members are associated with multiple cancer-related pathways, with a prominent enrichment in regulating cell proliferation. Epiplakin expression was significantly higher in squamous cell carcinomas (389.94 ± 70.56) compared with basal cell carcinomas (70.39 ± 15.32) and intradermal nevi, while basal cell carcinomas showed a significant decrease compared with normal skin (p < 0.05). In non-melanoma skin cancers, epiplakin expression demonstrated a strong positive correlation with E-cadherin (*r* = 0.565, p < 0.001) and a weak positive correlation with N-cadherin (*r* = 0.329, p < 0.05). No significant correlation was observed with Breslow thickness (p > 0.05).

**Study limitations:**

Retrospective design and the absence of high-grade squamous cell carcinoma cases in the study population.

**Conclusions:**

This is the first study to assess epiplakin expression among epithelial cutaneous cancers. Epiplakin appears to be associated with epithelial–mesenchymal transition and early tumor progression, and its differential expression pattern may provide diagnostic utility.

## Introduction

Carcinogenesis is a complex biological process involving cellular proliferation, invasion, and alterations in adhesion, with the cytoskeleton playing a pivotal role in maintaining cell shape, polarity, and mechanical stability. Among the proteins that anchor cytoskeletal elements ‒ such as microfilaments, intermediate filaments, and microtubules ‒ to cell junctions, the plakin family has emerged as essential for both tissue integrity and tumor biology. Members of this family, including Desmoplakin (DSP), Envoplakin (EVPL), Periplakin (PPL), Plectin (PLEC), and Bullous Pemphigoid Antigen-1 (BPAG1), provide structural links between cytoskeletal filaments and junctional complexes such as desmosomes and hemidesmosomes.^1^ Alterations in their expression or localization have been associated with various cancers, and some plakins have been proposed as potential biomarkers. For example, elevated serum anti-BPAG1 autoantibodies have been reported in melanoma patients compared with healthy individuals, although these findings have not been consistently replicated across studies.^2^, ^3^ Furthermore, studies in breast cancer have shown that decreased levels of plakins accompany early cytoskeletal disorganization. Their reduced expression leads to centrosome mispositioning and weakening of intercellular junctions ‒ changes that reflect loss of epithelial polarity and are characteristic of Epithelial-Mesenchymal Transition (EMT).^4^

Epiplakin (EPPK1) represents a less-characterized member of the plakin family, distinguished by its unique arrangement of tandem plakin repeat domains. Unlike other plakins, EPPK1 lacks spectrin repeats and a clear actin-binding domain, suggesting a divergent structural and functional role. While it is expressed in multiple epithelial tissues, its contribution to tumorigenesis remains incompletely understood, and studies on its involvement in cancer are still limited.^1^ Data from GEPIA2 indicate that EPPK1 mRNA levels are reduced in cutaneous melanoma, whereas UALCAN-based proteomic datasets show that protein-level alterations remain unclear.^5^, ^6^ Given that melanocytes and keratinocytes are interconnected through cadherin-based junctions, plakin proteins such as EPPK1 may contribute to the stabilization of these adhesion complexes. Clarifying whether EPPK1 expression varies between benign and malignant cutaneous lesions may offer diagnostic value.^7^ Despite this potential relevance, systematic studies examining EPPK1 expression across diverse cutaneous malignancies ‒ such as Squamous Cell Carcinoma (SCC) and Basal Cell Carcinoma (BCC) ‒ are currently lacking, and its expression profile in benign lesions like intradermal nevi has not been characterized either.

Previous studies have linked EPPK1 to the development of several cancer types, including hepatocellular, cervical, colorectal, bladder urothelial, and esophageal squamous cell carcinomas.^8^, ^9^, ^10^, ^11^, ^12^ Given that plakins contribute to cytoskeletal anchorage and junctional stability, altered EPPK1 expression may influence adhesion-related pathways that are also reflected in cadherin dynamics. Because the balance between E-cadherin and N-cadherin is an important indicator of adhesion loss and invasive potential, evaluating their relationship with EPPK1 expression may help clarify whether this plakin participates in adhesion remodeling in skin tumors. The present study aims to assess the immunohistochemical expression of EPPK1 alongside E-cadherin and N-cadherin in SCC, BCC, intradermal nevi, and normal skin specimens. Expression levels are compared across lesion types, and correlations with Breslow thickness are analyzed to explore associations with tumor invasiveness. Normal skin specimens serve as controls. Findings from this study may provide new insights into the diagnostic significance of EPPK1 and its potential interplay with cadherin-mediated adhesion in cutaneous lesions. In addition to the immunohistochemical evaluation, this study also aims to investigate the molecular interaction network of plakin family proteins and to identify their overlap with SCC-associated protein networks. Furthermore, GO and KEGG enrichment analyses are conducted to identify significantly enriched biological processes and signaling pathways associated with the overlapping proteins.

## Materials and methods

### Subjects and inclusion/exclusion criteria

This retrospective study analyzed skin tissue samples collected between 2021 and 2025 from the archives of the Pathology Department at Bilecik Education and 83 Research Hospital. A total of 24 intradermal nevi were included to allow comparison with 20 corresponding normal tissue samples in immunohistochemical analyses. In addition, 40 malignant cutaneous lesions were examined, comprising 20 BCCs and 20 SCCs.

Only primary cutaneous malignancies from patients who had not received prior radiotherapy or chemotherapy were included. Benign tissues obtained from patients with malignant lesions were excluded to avoid confounding. To minimize the influence of external factors on the analysis, individuals with known pre-existing dermatologic or pulmonary diseases were excluded, given emerging evidence that alterations in plakin-related proteins may affect keratinocyte adhesion and have been implicated in conditions such as bronchiolitis obliterans.^12^, ^13^, ^14^ To ensure adequate tissue for immunohistochemical assessment, only lesions measuring ≥ 0.5 mm were included. Ethical approval for this study was granted by the Ethics Committee of Bilecik University (approval nº 2025/7-12). All procedures were conducted in accordance with institutional guidelines and the principles outlined in the Declaration of Helsinki.

### Histopathological examination

Tissue specimens were fixed in 10% neutral buffered formalin to preserve structural integrity. Following fixation, samples underwent standard histopathological processing, including dehydration, clearing, and paraffin embedding. Sections of 4 µm thickness were prepared from paraffin blocks and mounted onto glass slides. Slides were deparaffinized in xylene and rehydrated through a graded ethanol series (100%, 90%, 80%, 70%). The sections were then stained with Hematoxylin and Eosin (H&E) to allow detailed visualization of cellular and tissue morphology. After staining, the slides were cover-slipped using Entellan.

Histopathological evaluation was performed using an Olympus CX23 brightfield microscope equipped with an Olympus EP50 camera (1920 × 1080 pixels). Lesions were categorized according to established diagnostic criteria: intradermal nevi were characterized by melanocytic nests confined to the dermis; BCC by basaloid tumor islands with peripheral palisading and hyperchromatic nuclei; and SCC by keratinocytic atypia, intercellular bridges, and keratinization.^15^, ^16^ For BCC subtyping, nodular, superficial, nodulocystic, and adenoid variants were classified as non-aggressive forms, whereas morpheaform and infiltrative subtypes were considered aggressive.^17^ Tumor invasion was assessed by measuring Breslow thickness, defined as the perpendicular distance from the granular layer of the epidermis to the deepest point of tumor extension.^18^

### Immunohistochemistry

Formalin-fixed, paraffin-embedded tissue blocks were sectioned at a thickness of 4 µm. Sections were deparaffinized in xylene and rehydrated through a graded alcohol series. Antigen retrieval was performed in citrate buffer (pH6), followed by blocking of endogenous peroxidase activity using 3% hydrogen peroxide.

The sections were incubated at room temperature with the primary antibodies: EPPK1 (1:100, PA5-64412, Thermo Fisher Scientific, Waltham, MA, USA), E-cadherin (1:250, sc-8426, Santa Cruz Biotechnology, Dallas, TX, USA), and N-cadherin (1:250, sc-59987, Santa Cruz Biotechnology, Dallas, TX, USA). After incubation with the appropriate secondary antibody, immunoreactivity was visualized using a streptavidin–HRP detection system, with 3,3′-Diaminobenzidine (DAB) as the chromogen. Slides were counterstained, dehydrated, and mounted for evaluation under a light microscope.

### Quantitative digital image analysis

Immunohistochemical staining intensity was quantified using ImageJ software (v1.53e, National Institutes of Health, USA). For each case, five regions of interest (ROIs) were randomly captured at ×400 magnification using an Olympus CX23 brightfield microscope equipped with an Olympus EP50 camera (1920 × 1080 pixels). Images were processed using ImageJ software (version 1.53e, National Institutes of Health, USA). To separate the chromogen signal from hematoxylin counterstaining, color deconvolution was applied, and the resulting image was converted to 8-bit grayscale for quantification. A fixed threshold limit, determined based on control staining to ensure appropriate discrimination of positive signal, was applied to all images to maintain consistency across samples. Immunoreactivity was quantified in terms of Integrated Optical Density (IOD), calculated as area × optical density.^19^ Mean IOD values obtained from each ROI were averaged and normalized (divided by 10⁶) prior to statistical analysis.

### Protein–protein interaction network analysis

A Protein–Protein Interaction (PPI) network was constructed for plakin family members, including EPPK1, DSP, EVPL, PPL, PLEC, BPAG1, and Microtubule Actin Crosslinking Factor-1 (MACF1), using a minimum confidence score of 0.4 and incorporating 100 additional interactors. Similarly, a disease-associated network related to SCC (DOID:1749) was generated using the same 0.4 confidence cutoff and 200 additional interactors. Both networks were generated and processed in Cytoscape v3.10 (Cytoscape Consortium, San Diego, CA, USA), where the Merge/Intersect tool was used to identify shared interactors. The resulting intersected protein set was subjected to KEGG pathway and GO functional enrichment analysis using the STRING v12 online platform. The top ten significantly enriched pathways (FDR < 0.05) were visualized as box plots, indicating both statistical significance and enrichment magnitude.^20^

### Statistical analysis

All statistical analyses were performed using SPSS Statistics v26 (IBM, Armonk, NY, USA). Data distribution was assessed for normality using the Shapiro-Wilk test. Differences in continuous immunohistochemical expression levels among groups were analyzed using the Kruskal-Wallis test, followed by Bonferroni-adjusted post hoc pairwise comparisons. Categorical variables were evaluated using the Chi-Square test. Correlations between protein expression levels and Breslow thickness were assessed with Spearman’s rank correlation analysis. A p-value < 0.05 was considered statistically significant.

## Results

### Clinico-pathological characteristics of the study samples

**Figure 1:**
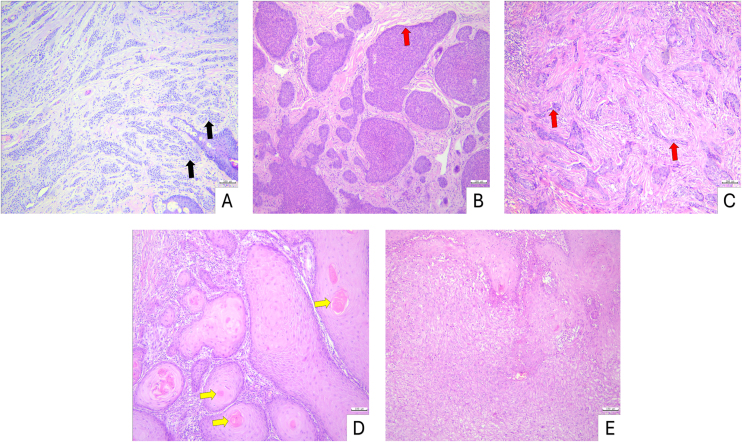
Histopathology of non-melanoma skin lesions (Hematoxylin & eosin stain) (A) Intradermal nevus, nests of round to oval nevus cells (black arrow) within the dermis. (B) Nodular BCC, basaloid nests with peripheral palisading (red arrow). (C) Infiltrative BCC, strands and cords of basaloid cells (red arrow) infiltrating the dermis. (D) Well-differentiated SCC, squamous nests with prominent keratinization and keratin pearls (yellow arrow). (E) Moderately differentiated SCC, squamous nests with intermediate keratinization. Scale bars: 100 µm.

Histopathological evaluation of intradermal nevi demonstrated nests of round to oval nevus cells dispersed throughout the dermis. BCC specimens exhibited basaloid cell nests extending into the dermis with prominent peripheral palisading. In SCC samples, infiltrative nests of squamous cells were observed, showing variable degrees of keratinization and occasional formation of keratin pearls (Fig. 1).

Clinico-pathological characteristics of the study samples are summarized in Table 1. The mean age differed significantly among the groups, with patients in the intradermal nevus group being younger (41.33 ± 10.33 years) compared to those in the SCC (69.05 ± 12.32 years) and BCC (70.40 ± 14.22 years) groups (p < 0.05). Gender distribution also varied significantly (χ^2^ = 7.612, p < 0.05), with a female predominance in the intradermal nevus group (66.7%) and a male predominance in the BCC group (75%). Tumor localization did not differ significantly among groups (p > 0.05), with the majority of lesions located on the head and neck in all groups. In SCC samples, histopathological grading was evenly distributed between low-grade (50%) and moderately differentiated (50%) tumors. In BCC, most cases were classified as non-aggressive (75%), while a smaller proportion were considered aggressive (25%). Breslow thickness did not differ significantly between SCC and BCC groups (4186.13 ± 2512.96 μm vs. 3534.26 ± 2652.04 μm, p > 0.05) (Table 1).

**Table 1:** Clinico-pathological parameters of intradermal nevi and non-melanoma skin cancers.

	Intradermal nevi	SCC	BCC	p-value
**Age (years)**	41.33 ± 10.33	69.05 ± 12.32	70.40 ± 14.22	**0.000^a,c^**
**Gender**				
Male (n)	8 (33.3%)	10 (50%)	15 (75%)	**0.022^b,c^**
Female (n)	16 (66.7%)	10 (50%)	5 (25%)	
Total %	24 (100%)	20 (100%)	20 (100%)	
**Localization**				
Head, Neck (n)	21 (59.4%)	18 (75%)	18 (75%)	0.953^b^
Trunk, Extremities (n)	3 (40.6%)	2 (25%)	2 (25%)	
Total (%)	24 (100%)	184 (100%)	184 (100%)	
**Grade**				
Low (n)		10 (50%)		
Mid (n)		10 (50%)		
Total %		20 (100%)		
**Types**				
Non-Aggressive (n)			15 (75%)	
Aggressive (n)			5 (25%)	
Total %			20 (100%)	
**Breslow (μm)**		4186.13 ± 2512.96	3534.26 ± 2652.04	0.372^a^

Data are shown as mean ± SD or number and percentage (%).

^a^ Kruskal-Wallis test; ^b^ Chi-Square test.

SCC, Squamous Cell Carcinoma; BCC, Basal Cell Carcinoma; SD, Standart deviation. Statistical significance (^c^ p < 0.05).

### EPPK1 expression across benign and malignant skin lesions

**Figure 2:**
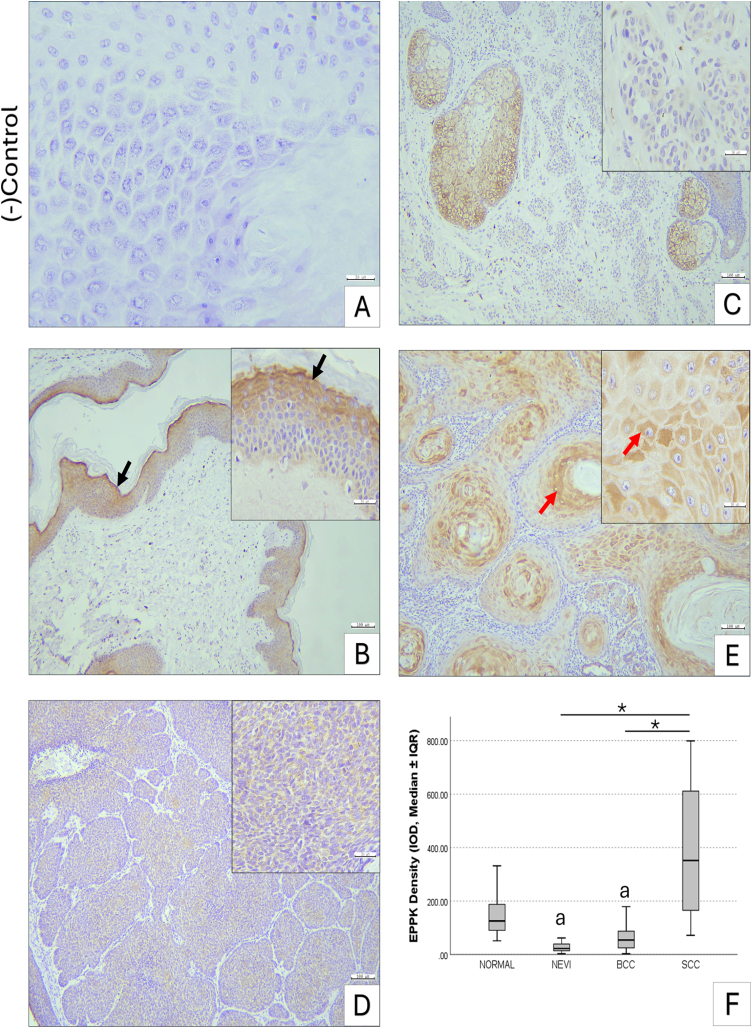
Immunohistochemical staining of EPPK1 in skin samples. (A) Negative control. (B) Normal skin, intense cytoplasmic EPPK1 expression in stratum granulosum (black arrow) and mild in stratum spinosum. (C) Intradermal nevus, very low cytoplasmic EPPK1 expression. (D) Basal cell carcinoma, moderate cytoplasmic EPPK1 expression. (E) Squamous cell carcinoma, strong cytoplasmic EPPK1 expression, especially in cells surrounding keratin pearls (red arrow). Scale bars: A, insets 30 µm, B–E 100 µm. (F) Boxplot showing EPPK1 optical density as median ± IQR; whiskers indicate minimum–maximum. Kruskal-Wallis test, * p < 0.05, a, Significantly different from normal skin and SCC. BCC, Basal Cell Carcinoma; SCC, Squamous Cell Carcinoma; IQR, Interquartile Range.

EPPK1 expression levels differed significantly among the groups. The intradermal nevus group (27.08 ± 3.82) exhibited markedly lower expression compared with normal skin (147.85 ± 17.58, p < 0.05) and the SCC group (392.66 ± 55.24, p < 0.05). In normal skin, EPPK1 immunoreactivity was predominantly cytoplasmic in cells of the stratum granulosum, with mild expression observed in the stratum spinosum. No significant difference in EPPK1 expression was noted between the nevus and BCC groups (70.39 ± 15.32, p > 0.05). Compared with normal tissue, BCC samples showed significantly lower EPPK1 expression (p < 0.05). In contrast, SCC specimens demonstrated significantly higher EPPK1 expression relative to both nevus and BCC samples (p < 0.05), while expression levels in SCC were not significantly different from the normal group (p > 0.05) (Fig. 2).

### Cadherin expression in non-melanoma skin cancer

**Figure 3:**
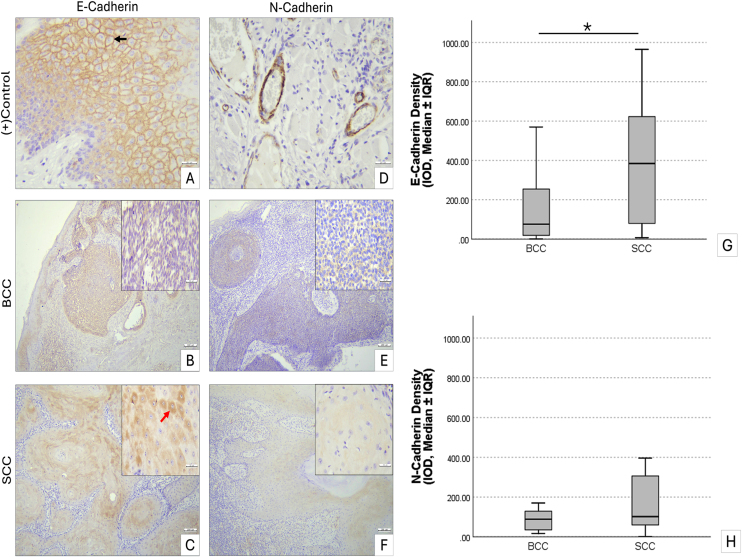
Immunohistochemical staining of E-cadherin and N-cadherin in skin samples. (A) Normal skin, E-cadherin predominantly at intercellular junctions with lower cytoplasmic expression. (B) BCC, E-cadherin cytoplasmic expression. (C) SCC, E-cadherin cytoplasmic expression, with increased intensity in some cells. (D) Normal skin, intense N-cadherin in vascular walls. (E) BCC, N-cadherin cytoplasmic expression. (F) SCC, low N-cadherin expression. Scale bars: A, D, insets 30 µm; B, C, E, F 100 µm. (G) Boxplot of E-cadherin optical density (median ± IQR), SCC significantly higher than BCC. (H) Boxplot of N-cadherin optical density (median ± IQR). Whiskers indicate minimum – maximum. Kruskal-Wallis test, * p < 0.05. BCC, Basal Cell Carcinoma; SCC, Squamous Cell Carcinoma; IQR, Interquartile Range.

E-cadherin expression differed significantly between the BCC and SCC groups. In normal skin, E-cadherin showed strong localization at intercellular junctions, with mild cytoplasmic staining in the squamous layer. The BCC group exhibited a mean E-cadherin optical density of 147.54 ± 38.26, whereas the SCC group showed markedly higher expression at 389.94 ± 70.56 (p < 0.05), with some cells exhibiting increased cytoplasmic staining. In contrast, N-cadherin expression was generally low in both BCC (139.61 ± 45.47) and SCC (216.37 ± 59.83) groups, with no statistically significant difference between them (p > 0.05) (Fig. 3).

**Figure 4:**
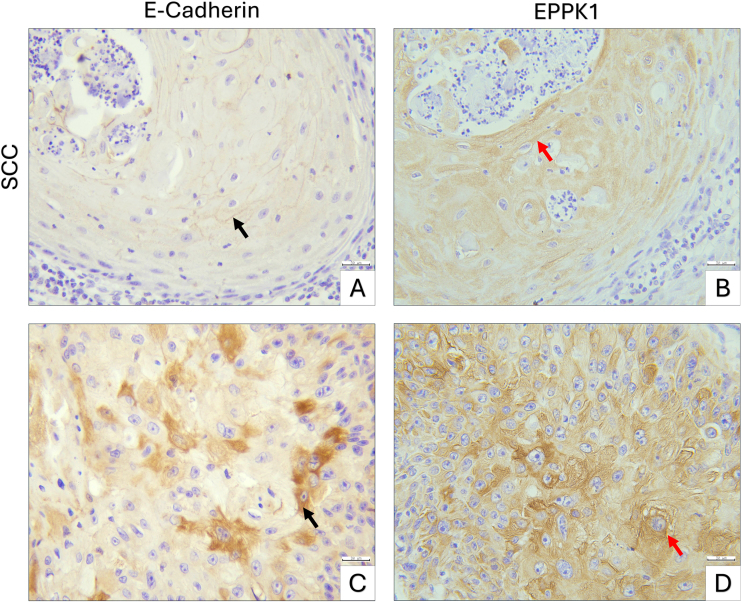
Immunohistochemical staining of E-cadherin and EPPK1 in SCC. (A‒B) Well-differentiated SCC, E-cadherin predominantly at intercellular junctions (black arrow) and corresponding region showing intense cytoplasmic EPPK1 expression (red arrow). (C‒D) Another area of the same SCC tumor, showing regionally increased cytoplasmic E-cadherin in some tumor cells (black arrow) and widespread cytoplasmic upregulation of EPPK1 across the same region (red arrow). Scale bars: 30 µm.

In well-differentiated SCC samples, E-cadherin immunoreactivity remained largely at intercellular junctions, whereas EPPK1 showed intense cytoplasmic expression. In areas where some tumor cells exhibited increased cytoplasmic E-cadherin, the corresponding sections displayed widespread cytoplasmic upregulation of EPPK1 (Fig. 4).

### Correlation of EPPK1 expression with cadherins and breslow thickness

**Figure 5:**
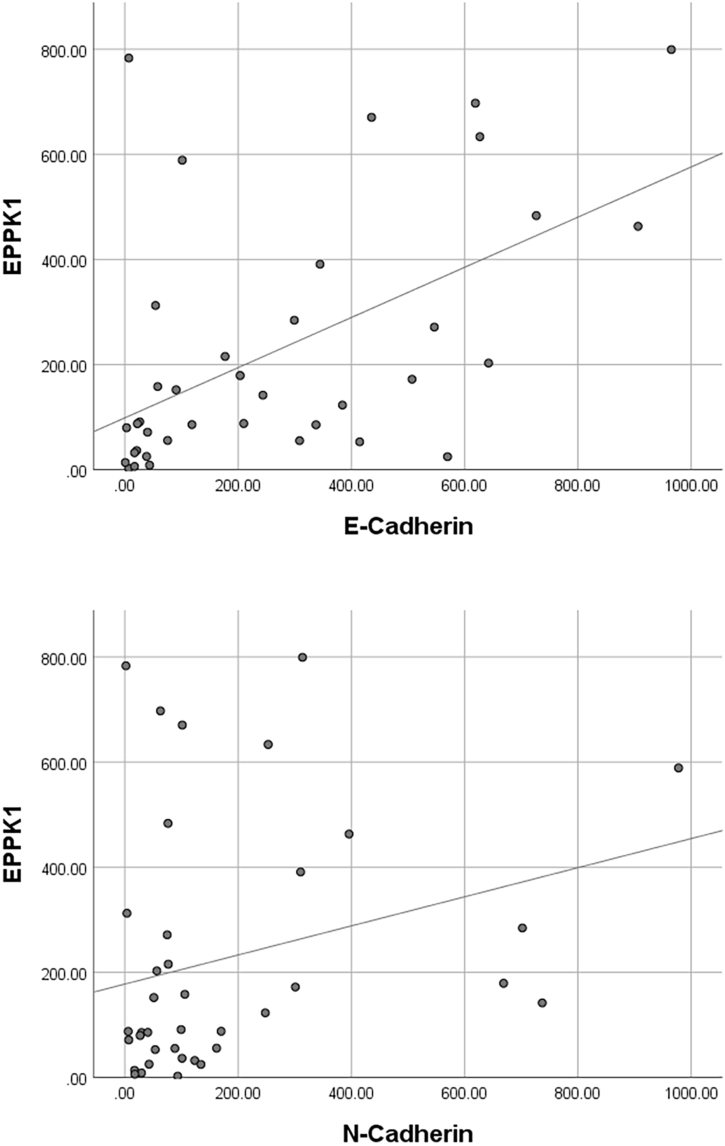
Scatter/dot graph of EPPK1 optical density versus E-cadherin and N-Cadherin optical density. Spearman's correlation test, p < 0.05.

Spearman correlation analysis demonstrated significant positive associations between EPPK1 expression and cadherin levels in non-melanoma skin cancers. EPPK1 exhibited a strong positive correlation with E-cadherin (*r* = 0.565, p < 0.001) and a moderate positive correlation with N-cadherin (*r* = 0.329, p < 0.05) (Table 2, Fig. 5). When tumor subtypes were analyzed separately, EPPK1 showed significant positive correlations with both E-cadherin and N-cadherin in BCC. In SCC, EPPK1 remained positively correlated with E-cadherin and additionally demonstrated a moderate positive correlation with Breslow thickness (p < 0.05), whereas no significant association was observed with N-cadherin (Table 2).

**Table 2:** Correlation analysis of EPPK1 expression with cadherins and Breslow thickness.

		Non-melanoma Skin Cancers	BCC	SCC
		EPPK1	EPPK1	EPPK1
**E-Cadherin**	*r*	**0.565**	**0.375**	**0.327**
	p	**0.0002^a^**	**0.011^a^**	**0.020^a^**
**N-Cadherin**	*r*	**0.329**	**0.350**	0.015
	p	**0.044^a^**	**0.018^a^**	0.917
**Breslow Thickness**	*r*	0.134	-0.224	**0.338**
	p	0.409	0.145	**0.015^a^**

Spearman's correlation test. BCC, Basal Cell Carcinoma; SCC, Squamous Cell Carcinoma; *r*, Correlation coefficient. ^a^ p < 0.05.

### Protein–protein interaction network analysis

PPI network associated with SCC (DOID:1749) was constructed using a minimum confidence score of 0.4 and including 200 additional interactors. The resulting network consisted of 246 nodes and 8258 edges, with an average node degree of 67.1 and an average local clustering coefficient of 0.692. In comparison, a random network of the same size was expected to contain 3172 edges, highlighting a significantly higher connectivity in the observed network (PPI enrichment p-value < 1.0e-16).

Intersection of the plakin family protein network with the SCC-associated network identified a subset of shared interactors. KEGG pathway enrichment analysis of these overlapped proteins showed significant enrichment (FDR < 0.05) across multiple pathways. The top ten enriched pathways were: MicroRNAs in cancer, Pancreatic cancer, Bladder cancer, Melanoma, Endocrine resistance, Proteoglycans in cancer, Prostate cancer, EGFR tyrosine kinase inhibitor resistance, Kaposi sarcoma-associated herpesvirus infection, and Non-small cell lung cancer (Table 3).

**Table 3:** KEGG enrichment analysis of the proteins shared between the plakin-family network and the Squamous Cell Carcinoma (SCC, DOID:1749) – associated network. Plakin family proteins (EPPK1, DSP, EVPL, PPL, PLEC, BPAG1, and MACF1) were merged with the SCC-associated PPI network, and overlapping proteins were subjected to KEGG pathway enrichment analysis using STRING v12. The table lists the top 10 most significantly enriched pathways. Each pathway entry also includes the number of associated genes (nGenes) and the specific pathway gene set identified. False Discovery Rate (FDR) < 0.05.

Enrichment FDR	nGenes	Pathway Genes	Signal	Pathways
2.20e-37	42	159	5.54	MicroRNAs in cancer
1.19e-27	27	71	5.25	Pancreatic cancer
4.14e-24	21	40	5.13	Bladder cancer
2.76e-26	26	72	4.98	Melanoma
1.26e-27	29	94	4.9	Endocrine resistance
1.81e-34	42	194	4.82	Proteoglycans in cancer
2.44e-27	29	97	4.81	Prostate cancer
9.41e-26	26	77	4.81	EGFR tyrosine kinase inhibitor resistance
1.10e-32	40	187	4.66	Kaposi sarcoma-associated herpesvirus infection
3.04e-24	24	68	4.66	Non-small cell lung cancer

EPPK1, Epiplakin; DSP, Desmoplakin; EVPL, Envoplakin; PPL, Periplakin; PLEC, Plectin; BPAG1, Bullous Pemphigoid Antigen-1; MACF1, Microtubule Actin Crosslinking Factor 1.

**Figure 6:**
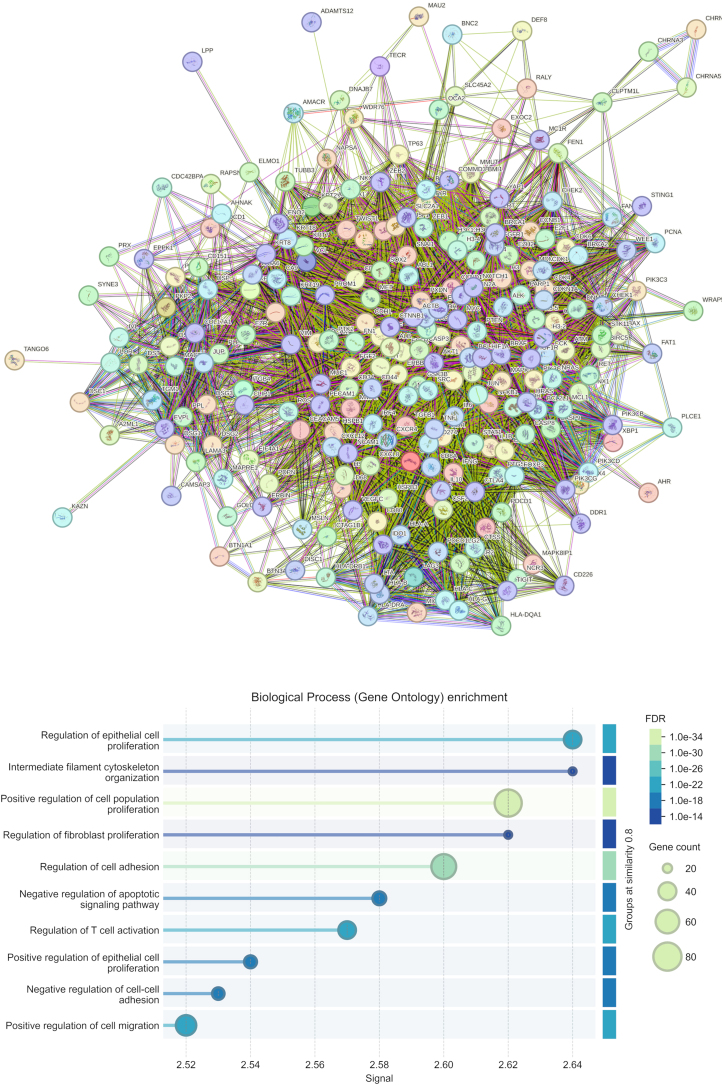
GO functional analysis of overlapped proteins from the intersection of plakin family proteins and squamous cell carcinoma associated networks. Plakin family proteins (EPPK1, DSP, EVPL, PPL, PLEC, BPAG1, and MACF1) were intersected with SCC-associated networks, and the shared proteins were subjected to KEGG pathway enrichment analysis. The box plot displays the ten most significantly enriched biological processes (FDR < 0.05). The color gradient from dark blue to pale green indicates decreasing FDR values, while the size of each box reflects the number of genes contributing to the enrichment, representing the weight of that pathway within the intersected dataset. EPPK1, Epiplakin; DSP, Desmoplakin; EVPL, Envoplakin; PPL, Periplakin; PLEC, Plectin; BPAG1, Bullous Pemphigoid Antigen-1; MACF1, Microtubule Actin Crosslinking Factor-1.

GO enrichment analysis identified significant overrepresentation of several biological processes. The most enriched terms included regulation of epithelial cell proliferation (FDR = 2.49E–21, 39 genes) and positive regulation of cell population proliferation (FDR = 1.91E–34, 75 genes). Additional proliferation-related processes were also highlighted, such as regulation of fibroblast proliferation (FDR = 7.42E–14, 18 genes) and positive regulation of epithelial cell proliferation (FDR = 1.32E–16, 27 genes). Cytoskeletal organization pathways were enriched, including intermediate filament cytoskeleton organization (FDR = 6.36E–14, 18 genes). Adhesion-associated processes showed significant enrichment, including regulation of cell adhesion (FDR = 1.65E–30, 65 genes) and negative regulation of cell–cell adhesion (FDR = 3.15E–16, 26 genes). Additional enriched terms included negative regulation of apoptotic signaling (FDR = 1.71E–17, 29 genes), regulation of T-cell activation (FDR = 5.95E–21, 39 genes), and positive regulation of cell migration (FDR = 9.71E–24, 48 genes) (Fig. 6).

## Discussion

The cytoskeleton and its associated junctional complexes play a central role in maintaining epithelial tissue integrity by regulating cell adhesion, polarity, and mechanotransduction ‒ processes that are profoundly altered during carcinogenesis. Plakin family proteins serve as critical structural bridges linking intermediate filaments to desmosomes and hemidesmosomes, thereby stabilizing epithelial architecture under physiological conditions.^1^ Accumulating evidence indicates that disruption of plakin-mediated cytoskeletal anchoring enhances cellular plasticity, promotes migratory capacity, and contributes to tumor progression across various malignancies. For instance, studies in ovarian cancer have shown that decreased PLEC and PPL expression in high-grade tumors coincides with reduced structural stability and a shift toward a more permissive state for invasion, even in the absence of overt EMT marker changes. These observations suggest that plakin loss may represent an early structural destabilization, priming tumor cells for migration by weakening epithelial anchorage.^21^ PPI network analysis of plakin family members in SCC revealed a highly interconnected network comprising 246 nodes and 8258 edges, with an average node degree of 67.1 and a clustering coefficient of 0.692 (PPI enrichment p < 1.0e16), highlighting a biologically meaningful organization. KEGG pathway enrichment of the overlapping proteins highlighted significant associations with multiple cancer-related pathways, including proteoglycans (FDR < 0.05). These results suggest that plakins function not only as structural components but may also participate in SCC signaling cascades that regulate tumor cell behavior, adhesion, and migration. Experimental evidence from HeLa cells shows that EPPK1 knockdown accelerates keratinocyte motility and induces cytoskeletal rearrangements, whereas overexpression suppresses motility.^22^

Among plakins, EPPK1 is distinctive in that it lacks spectrin repeats and a clear actin-binding domain, and its role in cancer remains poorly understood.^1^, ^23^ Research on EPPK1 is still limited, and its involvement in cutaneous malignancies has yet to be fully elucidated. Previous studies in esophageal SCC have reported markedly elevated EPPK1 expression compared with normal controls, whereas in colorectal adenocarcinomas, EPPK1 expression was decreased, suggesting a context-dependent role for this protein.^10^, ^12^ A recent review on head and neck cancers highlighted that desmosomal components, including plakin proteins, can paradoxically act, as their expression, localization, and interactions dynamically change during cancer progression.^24^ Likewise, although plectin is frequently upregulated in many tumor types, its downregulation has been reported in certain malignancies, emphasizing a dual expression pattern and underscoring the pivotal role of plakins in regulating tumor progression depending on cellular and tissue context.^25^ These observations suggest that the functional consequences of EPPK1 expression may vary according to tissue context and tumor type, highlighting the need for further investigation, particularly in cutaneous tumors. In cutaneous cancers, elevated serum anti-BPAG1 autoantibodies have been reported in melanoma patients compared with healthy individuals, indicating a potential, yet still uncertain, role of plakins as biomarkers.^2^, ^3^ In the present study, the authors aimed to systematically evaluate the immunohistochemical expression of EPPK1 in benign intradermal nevi, BCC, and SCC to clarify its potential role in skin tumor biology. The present findings demonstrated significantly higher EPPK1 expression in cutaneous SCC (392.66 ± 55.24) compared with BCC (70.39 ± 15.32) and benign intradermal nevi (27.08 ± 3.82) (p < 0.05). This variation likely reflects intrinsic cell type-dependent differences, as keratinocytic SCC cells possess distinct cytoskeletal architecture and keratinization-related features compared with BCC and benign melanocytic lesions, potentially contributing to their higher EPPK1 expression. Given this differential expression pattern, EPPK1 may have potential diagnostic utility in cutaneous malignancies. Routinely used markers, such as E-cadherin, p53, and certain metalloproteinases, have been suggested to aid in differentiating SCC from pseudocarcinomatous hyperplasia. However, a limitation of these studies was that marker expression was not analyzed according to tumor grade, and the differentiation of well-differentiated SCC from pseudocarcinomatous hyperplasia remained challenging.^26^ In this study, EPPK1 demonstrated a distinct expression pattern in low- to mid-grade SCC, suggesting that it may provide additional diagnostic value. Notably, in well-differentiated SCC regions, E-cadherin immunoreactivity remained largely at intercellular junctions, while EPPK1 showed intense cytoplasmic expression. In areas where some tumor cells exhibited increased cytoplasmic E-cadherin, EPPK1 expression was already widespread, indicating that changes in EPPK1 may precede detectable alterations in E-cadherin localization.

In normal skin, EPPK1 expression was predominantly observed in the suprabasal layers of the epidermis, with the most intense immunoreactivity localized near the granular layer, suggesting a role in late keratinocyte differentiation and epidermal barrier organization. In SCC samples, EPPK1 expression was particularly prominent in tumor nests and was most intense in squamous cells surrounding keratin pearls. This distinct spatial distribution may indicate that EPPK1 expression in SCC is associated with areas of keratinization and squamous differentiation. Such spatial patterns align with findings from epithelial models showing that keratin intermediate filaments and plakin family cytolinkers cooperatively stabilize keratin-rich surface structures, supporting the idea that plakins may influence keratinization dynamics in human epidermal lesions.^27^ Beyond malignancy, EPPK1 has also been implicated in epithelial barrier regulation. In psoriasis, EPPK1 is specifically downregulated in an interferon-γ–dependent manner, and its deficiency has been associated with impaired epithelial adhesion and barrier-related gene expression, supporting a role for EPPK1 in maintaining epithelial stability.^28^ Consistent with this concept, in the non-melanoma skin cancer group, EPPK1 expression showed a significant positive correlation with E-cadherin, suggesting that EPPK1 may be functionally linked to cadherin-mediated adhesion dynamics in cutaneous epithelial lesions.

Plakin family members can exert distinct and sometimes opposing effects in tumor biology. Notably, DSP displays a consistent tumor-suppressive pattern, with marked reductions observed across oral and lung carcinomas. This loss has been linked to poorer clinical outcomes, and experimental data demonstrate that DSP depletion enhances keratinocyte proliferation and activates prosurvival ERK/Akt signaling, whereas its overexpression suppresses lung cancer cell growth via modulation of Wnt pathway mediators. In contrast, the plakin-related protein MACF1 exhibits a more oncogenic profile, being highly expressed in glioblastoma; its knockdown reduces proliferation and migration while downregulating Wnt pathway components.^23^ Regarding EPPK1, previous studies in esophageal SCC reported functional knockdown experiments revealing reductions in cell proliferation, colony formation, migration, and invasion.^10^ Similarly, in adenocarcinomas such as colorectal cancer, EPPK1 expression was positively correlated with Ki67, suggesting a role in cellular proliferation.^12^ In line with these findings, the GO functional enrichment analyses demonstrated that the shared protein network formed by plakin family members in SCC was predominantly associated with cell proliferation–related processes, including “regulation of epithelial cell proliferation”, “positive regulation of cell population proliferation”, and “regulation of fibroblast proliferation”, all enriched with highly significant FDR values (FDR < 0.05). Beyond proliferative signaling, the network also showed strong associations with cytoskeletal organization, especially “intermediate filament cytoskeleton organization”, consistent with the canonical structural roles of plakins. Interestingly, reflecting the known dual and sometimes opposing functions of plakin proteins, the GO analysis revealed that the overlapping proteins were enriched in both positive and negative regulation of cell adhesion, highlighting their context-dependent contribution to junctional stability or loosening. Furthermore, enrichment in “positive regulation of cell migration” and processes linked to apoptotic signaling underscores a potential involvement of plakins in pathways that facilitate tumor cell motility and survival during carcinogenesis. In the context of the present study, these functional signatures guided the investigation of EPPK1 expression in cutaneous malignancies, prompting us to examine its associations with EMT markers and Breslow thickness to better understand its potential contribution to tumor progression.

EMT involves the loss of cell–cell junctions and epithelial polarity, including adherens junctions. Cadherins, as adhesion molecules, can also act as signaling mediators, influencing cellular behaviors such as migration, proliferation, apoptosis, and differentiation.^29^ Pogorzelska-Dyrbuś et al. reported significantly higher E-cadherin and N-cadherin expressions in SCC, with N-cadherin levels being significantly elevated compared with BCC, which was associated with a relatively higher metastatic potential.^30^ Similarly, Kim et al. observed increased vimentin expression in SCC.^31^ In the present study, although SCC samples exhibited higher N-cadherin levels (216.37 ± 59.83), the difference compared with BCC (139.61 ± 45.47) was not statistically significant (p > 0.05), likely due to the predominance of low- and mid-grade SCCs in the studied cohort. Suiqing et al. reported that E-cadherin expression is markedly lower in poorly differentiated SCC compared with well-differentiated tumors.^32^ Consistently, in the present study, E-cadherin showed intense cytoplasmic immunoreactivity in SCC cases (389.94 ± 70.56, p < 0.05). In the literature, this shift of cadherins from the cell membrane to the cytoplasm is often interpreted as a functional loss of these adhesion molecules, potentially compromising cell integrity and facilitating malignant transformation and metastasis during EMT.^29^ Interestingly, EPPK1, which exhibited a positive correlation with E-cadherin in the non-melanoma skin cancer samples, may similarly be involved in EMT-related or other cellular processes, including proliferation. Its immunoreactive localization with enhanced cytoplasmic expression was observed particularly in nests of squamous cells surrounding keratin pearls. The positive moderate correlation between EPPK1 expression and Breslow thickness in SCC may partly reflect increased keratinization and the more prominent formation of keratin pearls in thicker tumors, given the structural association of EPPK1 with the cytoskeletal network.

In lung adenocarcinoma, EPPK1 knockdown led to increased E-cadherin expression and a concomitant decrease in vimentin levels, suggesting a role in modulating epithelial–mesenchymal characteristics.^33^ In corneal epithelial wound healing models, EPPK1 deficiency was associated with decreased expression of E-cadherin, keratin-6, and vimentin, indicating a role in cytoskeletal regulation and potentially facilitating cell migration during tissue repair.^34^ In the present study, EPPK1 expression in non-melanoma skin cancers showed a strong positive correlation with E-cadherin (*r* = 0.565, p < 0.001) and a modest positive correlation with N-cadherin (*r* = 0.329, p < 0.05). Lopes et al. reported a positive correlation between N-cadherin and E-cadherin in melanoma, interpreted as a partial EMT state with a hybrid cadherin expression profile.^35^ Venza et al. found that reduced E-cadherin in cutaneous melanoma did not significantly correlate with clinical stage or Breslow thickness, and they suggested that its downregulation may be more closely associated with regulating melanoma cell proliferation.^36^ In the non-melanoma skin cancer samples, the low correlation between EPPK1 and N-cadherin, along with the absence of a significant correlation with Breslow thickness, indicates that EPPK1 alone may not serve as a reliable invasion marker, although it could play a role in the early stages of EMT.

Epidemiologically, intradermal nevi are predominantly observed in female patients, with 80.46% of cases reported in women.^37^ A retrospective analysis of cases diagnosed between 2010 and 2018 similarly confirmed this female predominance, with 1973 women and 667 men affected.^38^ In contrast, BCC and SCC primarily affect older adults, with the most frequent age of onset around 70–85 years.^39^ Specifically, cutaneous SCC generally presents around 70-years of age, with over 80% of cases occurring in individuals aged 60 or older.^40^ Consistently, in the present study, SCC and BCC groups exhibited a higher proportion of males and an increased mean age compared with the intradermal nevus group, reflecting known epidemiological trends.

The present study represents the first systematic evaluation of EPPK1 expression in the context of cutaneous malignancy progression. Although limited by its retrospective design, the findings provide valuable insights into the potential role of EPPK1, particularly in relation to EMT processes. Notably, EPPK1 expression was significantly higher in well-differentiated SCC compared with BCC and benign intradermal nevi, and it demonstrated a positive correlation with E-cadherin, suggesting a role in EMT dynamics and potentially other early tumor-related processes.

In conclusion, EPPK1 appears to act as a context-dependent molecule associated with EMT, showing elevated expression in keratinocytic SCC. Its differential expression pattern and correlation with key adhesion markers highlight its potential utility as a diagnostic biomarker for cutaneous malignancies. Future studies with larger cohorts, including high-grade SCCs, are warranted to further clarify the prognostic significance of EPPK1.

## ORCID ID

Özlem Türelik: 0000-0001-6057-9171

## Institutional review board statement

Ethical approval was obtained from the Ethics Committee of Bilecik University (approval number: 2025/7-12, Date: August 04, 2025). All methods were conducted in accordance with the ethical standards of the institutional research committee and with the Declaration of Helsinki.

## Research data availability

The entire dataset supporting the results of this study was published in this article.

## Financial support

None declared.

## Authors' contributions

Damla Gül Fındık: Study conception and planning; data collection, analysis and interpretation; statistical analysis; preparation and writing of the manuscript; approval of the final version of the manuscript.

Özlem Türelik: Data collection, analysis and interpretation; preparation and writing of the manuscript; approval of the final version of the manuscript.

## Conflicts of interest

None declared.
